# Comparison of malaria treatment outcome of generic and innovator’s anti-malarial drugs containing artemether–lumefantrine combination in the management of uncomplicated malaria amongst Tanzanian children

**DOI:** 10.1186/s12936-019-2769-z

**Published:** 2019-04-11

**Authors:** Manase Kilonzi, Omary Minzi, Ritah Mutagonda, Philip Sasi, Appolinary Kamuhabwa, Eleni Aklillu

**Affiliations:** 1 Department of Clinical Pharmacy and Pharmacology, School of Pharmacy, Muhimbili University of Health and Allied Sciences, P. O. BOX 65013, Dar es Salaam, Tanzania; 2 Department of Clinical Pharmacology, School of Medicine, Muhimbili University of Health and Allied Sciences, P. O. BOX 6515, Dar es Salaam, Tanzania; 3 Division of Clinical Pharmacology, Department of Laboratory Medicine, Karolinska Institutet, Karolinska University Hospital-Huddinge C1:68, 141 86 Stockholm, Sweden

**Keywords:** Artefan^®^, Coartem^®^, Effectiveness

## Abstract

**Background:**

In 2006, artemether–lumefantrine (ALU), specifically Coartem^®^ (Novartis Pharma AG, Basel Switzerland), was approved as the first-line drug for treatment of uncomplicated malaria in Tanzania. Due to poor availability and affordability of the innovator’s product, the government of Tanzania in 2013 prequalified the use of generic anti-malarial drugs, whereby Artefan^®^ (Ajanta, Pharma Ltd, India) was the first to be approved.

**Methods:**

This was an equivalence prospective study that aimed to determine the effectiveness of anti-malarial generic Artefan^®^ in comparison with innovator’s product Coartem^®^. Patients aged 6 to 59 months with uncomplicated malaria were recruited and randomized to either receive Artefan^®^ or Coartem^®^ as a control. Participants were required to revisit clinic five times as follow up to monitor treatment outcome as per World Health Organization recommendations. On each visit, thick and thin blood smears, dried blood spot (DBS), haemoglobin concentrations and auxiliary temperature were performed and documented.

**Results:**

Out of 230 recruited participants, 200 met inclusion criteria and were randomized equally to receive Artefan^®^ and Coartem^®^. The overall PCR uncorrected cure rate were 80% for Artefan^®^ and 75% for Coartem^®^ (p = 0.44). Adequate clinical and parasitological response were 82.1% for Artefan^®^ and 74.7% for Coartem^®^, and there was no early treatment failure (ETF) observed in both arms of treatment. Both drugs showed excellent early parasite clearance, whereby no participants had peripheral parasitaemia on day 3. Late clinical failures (LCF) were 3.6% for Artefan^®^ and 1.3% for Coartem^®^ (p = 0.31), and late parasitological failure (LPF) were 15.4% for Artefan^®^ and 22.7% for Coartem^®^ (p = 0.32). Mean haemoglobin (g/dl) concentrations observed on day 28 were higher compared to day 0 for both drugs, although not statistically significant. Only one (1.3%) participant on Artefan^®^ had temperature ≥ 37.5 °C on day 3.

**Conclusion:**

The findings of this study indicate that both Artefan^®^ and Coartem^®^ are equivalent and effective in the management of uncomplicated malaria amongst children in the Coast part of Tanzania.

## Background

Malaria continues to be one of the major public health problems in many regions of the world, particularly the developing countries [1]. African region accounts for about 90% of global malaria cases and deaths, in which majority are from Sub-Saharan African (SSA) countries [2] (carries about 80% of the global malaria burden).

A majority (90%) of Tanzanians (both mainland and Zanzibar) live in moderate to high malaria endemic region [2]. About 10 to 12 million people in Tanzania contact malaria every year and 80,000 of them die, in majority children. *Anopheles gambiae* and *Anopheles arabiensis* are the two species of Anopheles mainly responsible for malaria transmission in Tanzania. Ninety-six percent of malaria in Tanzania is caused by *Plasmodium falciparum* and the remaining 4% due to *Plasmodium malariae* and *Plasmodium ovale* [2]. Co-existing of different species of *Plasmodium* determine appropriate treatment approach [3] and is one of the criteria to be considered in the development and review of guidelines.

Proper diagnosis and prompt treatment with effective anti-malarial drugs are one of the major tools in the control of malaria. The emergence of resistance of *P. falciparum* to anti-malarial drugs has led to changes of drugs from chloroquine to sulfadoxine–pyrimethamine (SP) and currently artemisinin-based combination therapy (ACT) [4, 5]. In 2006, ACT was introduced in Tanzania and artemether–lumefantrine (ALU) was accepted as the first-line and dihydroartemisinin–piperaquine (DHA-P) as the second-line for management of uncomplicated malaria in both adult and children [6]. Since the introduction of ALU, the innovator’s product Coartem^®^ (Novartis Pharma AG, Basel Switzerland) was prequalified by the Tanzanian government and the product was available in all public health facilities. Due to high cost and poor availability of the innovator’s product, the government of Tanzania prequalified the use of generic ALU, which is now more widely available in public health facilities than Coartem^®^. Artefan^®^ (Ajanta, Pharma Ltd, India) was the first generic drug to be prequalified by the government of Tanzania and is still available in the supply chain.

For the purpose of ensuring good performance and detection of emergence of resistance of anti-malarial drugs, especially those used as a first-line and second-line in a country, the World Health Organization (WHO) recommends frequent monitoring of their effectiveness. Early parasite clearance after initiation of ACT is considered to be an indicator of treatment outcome [7, 8]. Peripheral parasitaemia on day 1, day 2 and day 3 following initiation of ACT can be used to assess treatment outcome in individuals with uncomplicated malaria [4]. Peripheral parasitaemia on day 1 and 2 or parasite density on day 3 of > 25% parasite density of day 0, is regarded as early treatment failure. Moreover, early parasite clearance is used as an indicator of *P. falciparum* resistance to artemisinin. Proportion of patients with detectable parasitaemia on day 3, 72 h following initiation of anti-malarial therapy is used to predict resistance (i.e. if 10% of the patients have peripheral parasitaemia on day 3 is regarded to be a sign of *P. falciparum* resistance to artemisinin [5, 9]. Adequate clinical and parasitological response (cure rate) is mainly defined by the absence of peripheral parasitaemia on day 28.

Generic artemisinin-based combinations play a great role in the management and control of malaria in developing countries as they are highly affordable and readily available. However, availability of substandard anti-malarial drugs in SSA, including Tanzania, has been reported and they pose a threat to the gained successes in malaria control [7, 8]. Poor quality anti-malarial drugs expose parasites to a sub-therapeutic drug pressure, thus providing windows for parasite selection, treatment failures and spread of tolerance/resistance and may also be a threat to patients’ safety [8, 10]. In 2012, Minzi et al. reported compliance of bioequivalence criteria of Artefan and Coartem in relation to AUC and Cmax, as per FDA recommendations but the generic product could not meet the 95% confidence interval bioequivalence criterion leaving a room of doubt on the effectiveness of the generic product [11]. The objective of this study was to compare malaria treatment outcome of Artefan and Coartem in the management of uncomplicated malaria amongst Tanzanian children.

## Methods

### Study design, site and population

This was an equivalence prospective study that aimed to determine the effectiveness of anti-malarial generic Artefan in comparison with innovator’s product Coartem. The study was conducted at Kibiti health centre in Kibiti district, which is an area in the coastal region of Tanzania, with a malaria prevalence of about 10.2%. In this surveillance of anti-malarial drugs, a formula for sample size calculation in equivalence studies [12] was used to obtain the estimated sample size. One side type 1 error of 2.5% (confidence level of 95%), power of 90%, equivalence limit of 0.55 and a maximum of 10% difference of cure rates between treatment groups was considered equivalent. A minimum sample size of 93 was required for each treatment arm. In addition, a 20% loss to follow was added, thus making the sample size of 116 participants for each treatment arm.

Consecutive sampling technique was used to enroll patients aged between 6 and 59 months with uncomplicated malaria (screening for malaria positivity was done by malaria Rapid Diagnostic Tests (RDT) after being examined clinically by physician). After being enrolled, patients were randomized to receive either the generic artemether–lumefantrine (Artefan) or the innovator’s product (Coartem) as a control group, and thereafter followed until day-28 as per WHO guidelines [13]. Eligibility criteria were (1) aged 6–59 months, (2) uncomplicated malaria, (3) mono-infected with *P. falciparum*, (4) agreement to come to the study clinic for any febrile episode or other illnesses with the parent/guardian, (5) residence in Kibiti town perimeter, (6) absence of chronic diseases like HIV/AIDS, kidney disease and any active medical problem requiring hospitalization, and (7) provision of informed consent form signed by parents/guardians.

### Malaria diagnosis and treatment

Diagnosis of malaria followed current Tanzania standard treatment guidelines (STG) in which uncomplicated malaria is defined as symptomatic malaria without signs of severity or evidence (clinical or laboratory) of vital organ dysfunction (Fever, Headache, Joint pains, Malaise, Vomiting/diarrhoea, Body ache, Body weakness, Poor appetite, Pallor, Enlarged spleen). But the clinical features listed above are not specific for malaria and can be found in several other febrile conditions. Therefore, diagnosis of uncomplicated malaria was done by using RDT (CareStart™ Malaria HRP2 (pf) (Access Bio, Ethiopia)) followed by confirmation by microscopy. Children who were malaria positive aged between 6 and 59 months were randomized to receive either Artefan or Coartem, as recommended by malaria treatment guidelines [14]. Both Artefan and Coartem are fixed combination of 20 mg artemether and 120 mg lumefantrine in a tablet. Study drugs used were of the same butch number and were purchased from a registered market authorization holder (MAH) in Dar es Salaam, Tanzania. Dosing was done per weight (kg) of the patients according to the manufacturer’s recommendation which was the same for both drugs. The full course of treatment for all study patients consists of 6-doses given twice daily for 3 days. The first daily dose of study was given under direct observation at the study clinic and the remaining doses were given to study participants’ parents/guardians and were administered at home. Parents/guardians were asked to bring back the children on day 3, 7, 14, 21 and 28 as follow up. On day 3 parents/guardians were asked to come with the blister pack as a way of ensuring adherence to the medication. Episodes of severe malaria and recurrent malaria occurring within 14 days of therapy were treated with artesunate injection.

### Laboratory investigation

Thick and thin blood smears were stained with 2% Giemsa for 30 min and microscopic examination was performed by trained laboratory technologists who were not involved in direct patient care. Parasite densities were calculated from thick blood smears by counting the number of asexual parasites per 200 leukocytes (or per 500 leukocytes, if the count was < 10 asexual parasites/200 leukocytes), assuming a leukocyte count of 8000/μl. A blood smear was considered negative when the examination of 100 high power fields did not reveal asexual parasites. For quality control, all slides were read by two readers. In case of discordant readings, an independent third reader was used settled discrepancies between the first and second readings. Laboratory technicians were blinded to the study participants’ treatment assignments. Thin smears were used to determine the parasite species [4, 15].

Haemoglobin (Hb) was determined on D0, D3, D7, D14 and D28 and quantification of Hb was done using a Hemocue Hb 201^+^ (Angelholm, Sweden) microcuvette machine, from. The drop of blood was collected in an Hb 201 microcuvette and read using HemoCue Hb 201^+^ device and result was recorded in g/dl.

### Statistical methods

Data were entered into an Excel sheet and analysis was done by using Statistical Package for Social Sciences (SPSS) software version 20 (SPSS Inc., Chicago, IL, USA). Mean, geometric mean, range, standard deviation and percentage were used to summarize results. Chi square was used in finding association between variables of interest. Kaplan–Meier using per-protocol approach was used to determine cure rate and malaria recurrent rate. p-value of < 0.05 was considered to be statistically significant.

## Results

**Fig. 1:**
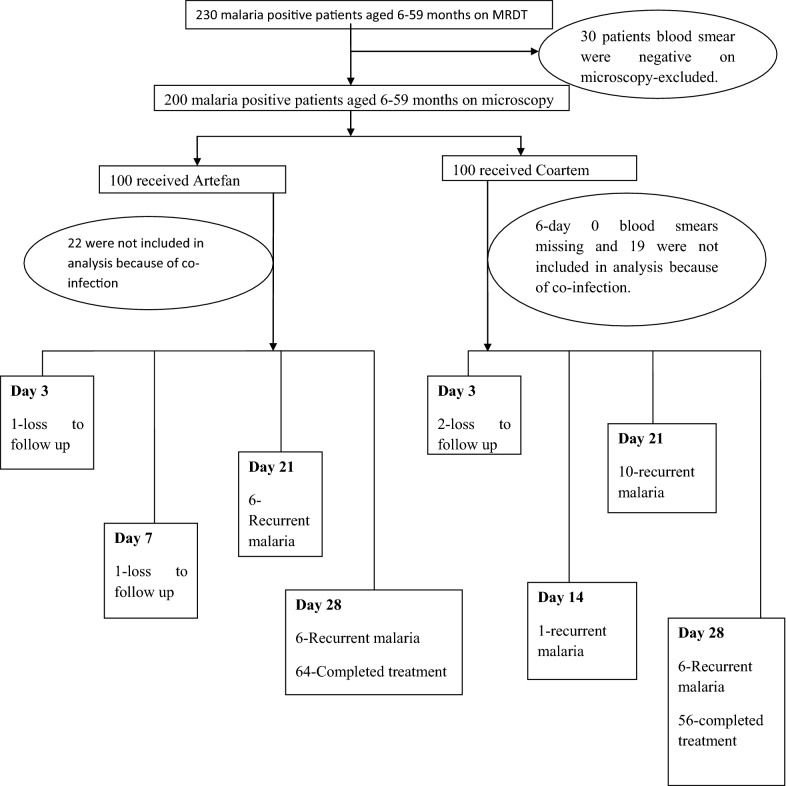
Flowchart of study enrollment and follow-up procedure

Two hundred and thirty patients aged 6–59 months with uncomplicated malaria were recruited to participate in this study. All study participants were positive on RDT and 30 patients were excluded because blood smear were found to be negative on microscopy. Two hundred patients were then randomized for treatment, whereby 100 received Artefan and another 100 Coartem. The study flowchart indicating enrolment and follow up procedures is presented in Fig. 1. Twenty-two children on Artefan and 19 on Coartem were excluded during analysis because they had co-infection of *P. falciparum* and *P. malariae* (Fig. 1).

### Baseline characteristics of study participants

Majority of patients were females (54%) on Artefan and male (51.6%) on Coartem with mean age of 33 months and 32 months, respectively. Baseline mean temperature was 38.4 °C on Artefan and 38.5 °C on Coartem. Baseline peripheral parasitaemia geometric means were 10,074**/**µl in Artefan and 9320**/**µl in Coartem arms, while mean baseline Hb concentrations were 9.2 g/dl and 9 g/dl, respectively. Before initiation of treatment, 22% of children on Artefan and 20.2% on Coartem had co-infection of *P. falciparum* and *P. malariae*. (Table 1). There were no significant differences in the baseline characteristics of study participants between the two study groups. By using mid upper arm circumference (MUAC), majority of patients had good nutritional status in both arms of treatment.

**Table 1:** General characteristics of study participants

Characteristics	Treatment arm		p-value
	Artefan^®^ (n = 78)	Coartem^®^ (n = 75)	
Age in mean (range)	32.74 (50-60)	32.17 (47–60)	0.81
Female n (%)	42 (53.8%)	33 (44%)	0.22
Temperature (°C) in mean (SD)	38.4 (1.13)	38.5 (1.18)	0.60
Parasite density (parasite/μl) in mean (SD)	9730.93	8745.75	0.16
Hemoglobin (g/dl) in mean (SD)	9.3 (1.5)	9.3 (1.8)	0.88
Good nutritional status (based on MUAC)	71 (92.2%)	68 (91.9%)	0.94

### Treatment outcome

The treatment outcomes were defined as: early treatment failure (ETF)—presence of danger signs or complicated malaria with a positive blood smear on day 3 of treatment, or day 3 parasite density > 25% of day 0 parasite density, or positive blood smear on day 3 with auxiliary temperature ≥ 37.5 °C. Late clinical failure (LCF)—danger signs or severe malaria in the presence of parasitaemia on any day between day 4 and day 28 in patients who previously did not meet any of the criteria of early treatment failure, and presence of parasitaemia on any day between day 4 and day 28 with auxiliary temperature ≥ 37.5 °C in patients who previously did not meet any of the criteria of early treatment failure. Late parasitological failure (LPF)—presence of parasitaemia on any day between day 7 and day 28 (day 42) with auxiliary temperature < 37.5 °C in patients who previously did not meet any of the criteria of early treatment failure or late clinical failure. Adequate clinical and parasitological response (ACPR)—absence of parasitaemia on day 28 irrespective of auxiliary temperature, in patients who previously did not meet any of the criteria of early treatment failure, late clinical failure or late parasitological failure.

**Fig. 2:**
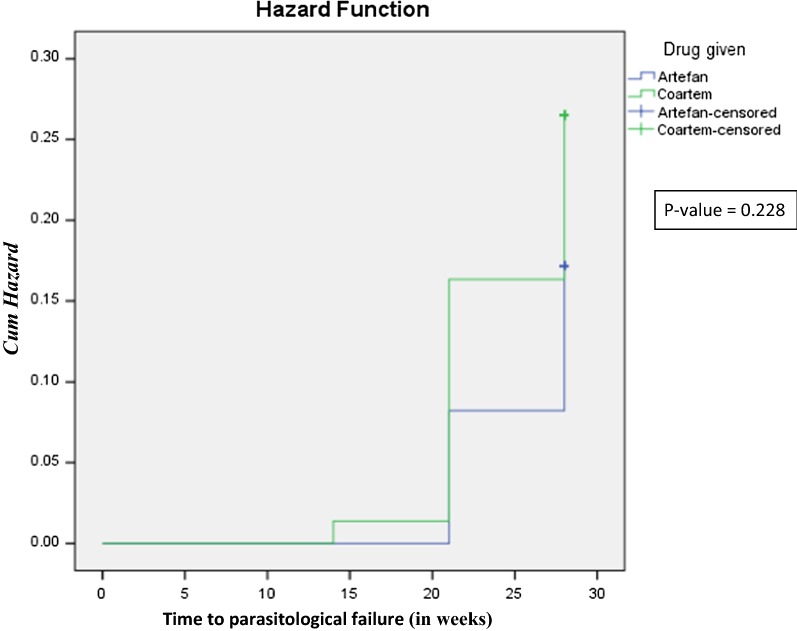
Kaplan Meier curve showing cumulative hazard proportion of children to get recurrent parasitaemia during follow up after being treated with either Artefan^®^ or Coartem^®^

None of the study participants had peripheral parasitaemia on day 3 on both Artefan and Coartem treatment arms. On day 3 only, 1 (1.1%) patient on Artefan treatment arm had temperature above 37.5 °C. Slightly decrease in Hb concentration was observed on day 3 in both arms of treatment but the difference was not statistically significant (Table 2). The mean Hb concentrations on day 28 were 10.3 ± 1.4 g/dl in Artefan and 10.4 ± 1.3 g/dl in Coartem, to some extent high compared to day 0. No early treatment failure (ETF) was observed on day 3 on both treatment arms. Three (3.6%) patients in Artefan and 1 (1.3%) in Coartem treatment arms were classified as late clinical failure (LCF). Fourteen (16.7%) patients in Artefan and 16 (21.3%) in Coartem treatment arms were classified as late parasitological failures (LPF), most of the failures were observed on day 21 and day 28. Adequate clinical and parasitological responses (ACPR) on day 28 were 70 (83.3%) patients in Artefan and 58 (77.3%) in Coartem treatment arms, but the difference in ACPR was not statistically significant (p = 0.34) between the two drugs (Table 3). Children in the Coartem (27%) arm had high risk to get recurrent malaria compared to those in the Artefan (17%) arm, but the difference was not statistically significant (p = 0.23) (Fig. 2).

**Table 2:** Temperature persistence and parasite clearance

Characteristics	Treatment outcome		p-value
	Artefan^®^ arm (n = 78)	Coartem^®^ arm (n = 75)	
Temperature persistence (temperature ≥ 37.5 °C)			
On day 0	66 (84.6%)	61 (81.3%)	0.60
On day 3	1 (1.3%)	0 (0.0%)	0.54
On day 7	2 (2.9%)	0 (0.0%)	0.25
On day 14	2 (2.9%)	0 (0.0%)	0.26
On day 21	1 (1.6%)	0 (0.0%)	0.51
On day 28	3 (5%)	1 (1.7%)	0.31
Parasite clearance			
Smear positive on day 3	0	0	
Smear positive on day 7	0	0	
Smear positive on day 14	0 (0.0%)	1 (1.1%)	0.71
Smear positive on day 21	6 (7.7%)	10 (13.3%)	0.42
Smear positive on day 28	6 (7.7%)	6 (8%)	0.97
Mean hemoglobin concentration (SD)			
Day 3	8.9 (1.5)	8.8 (1.4)	0.96
Day 7	9.0 (1.4)	9.1 (1.5)	0.73
Day 14	9.8 (1.3)	9.6 (1.3)	0.43
Day 21	10 (1.4)	9.8 (1.4)	0.97
Day 28	10.3 (1.4)	10.4 (1.3)	0.74

**Table 3:** Treatment outcome during 28 days follow up period

Treatment outcome	Treatment arm		p-value
	Artefan^®^ (n = 84)	Coartem^®^ (n = 75)	
Late clinical failure (LCF)	1 (3.6%)	1 (1.3%)	0.31
Late parasitological failure (LPF)	12 (15.4%)	17 (22.7%)	0.32
Adequate clinical and parasitological response (ACPR)	64 (82.1%)	56 (74.7%)	0.44

## Discussion

After the introduction of artemisinin-based combination therapy (ACT) as the first-line and second-line option in the management of uncomplicated and complicated malaria by the WHO together with other methods of malaria prevention such as insecticide-treated nets (ITN) and indoor spraying, prevalence of malaria has decline in many countries. Protecting the efficacy of ACT for the treatment for falciparum malaria is among the top global public health priorities. The presence of falsified and substandard drugs has been reported in many parts of the world, including sub-Saharan Africa (SSA) [16]. The WHO has identified anti-malarials as one group of drugs at highest risk of being falsified or sub-standard, because of high demand especially in moderate to high endemic regions. Previous studies reported the presence of poor quality and substandard anti-malarial drugs in the Tanzanian market [17, 18]. Regular surveillance and assessment of the performance of generic anti-malarials through therapeutic efficacy studies is of importance for malaria control.

In this study, the therapeutic efficacy of generic anti-malarial Artefan and innovator’s product Coartem were compared. Early parasite clearance was excellent for both drugs with no detected peripheral parasitaemia on day 3 (72 h, following initiation of therapy) using microscopy. Temperature clearance was also outstanding for both Artefan and Coartem, with only 1 (1.1%) patient in the Artefan arm having temperature above 37.5 °C on day 3. The early parasite clearance in this study on day 3 was the same as what was reported in Uganda, in which early parasite clearance following treatment with artemether-lumefantrine was excellent [4].

Resistance of *Plasmodium* species to artemisinin has been reported in eastern and southern Asian countries [19], but not yet in Africa. In the present study, none of the study participants treated with Artefan or Coartem had peripheral parasitaemia on day 3. This may indicate absence of resistant strains of *P. falciparum* to artemisinin in Tanzania. This is in line with the WHO 2009 anti-malarial protocol, that if 10% of the study participants have peripheral parasitaemia on day 3, it is an indicator of emergence of artemisinin resistance to *Plasmodium* species [13].

Early treatment failure (ETF) was not observed in both Artefan and Coartem treatment arms, whereas late clinical failures (LCF) were 3.6% and 1.3% in Artefan and Coartem arms, respectively. However, late parasitological failures (LPF) were high in both Artefan (15.4%) and Coartem (22.7%) arms. Artemisinin products (for this study artemether) have a short half-life. Since most of the LCF and LPF occurred on day 21 and 28 with only one LPF occurring on day 14, its means the partner drug (lumefantrine) offers a prolonged protection against malaria of two to 3 weeks. From the survival analysis, cumulative hazard proportion curve shows that children using Coartem might be at higher risk of parasitological failure compared to children using Artefan. The observed differences in late treatment failures (LCF and LPF) between Artefan and Coartem were not statistically significant, an indication that the generic anti-malarial Artefan is effective for management of uncomplicated malaria in Tanzanian children.

The observed LCF in this study is comparable to what was reported in Ethiopia [20]. In the Ethiopian study, LCF were 1.4% on day 21 and 2.8% on day 28. On the other hand, the results of LCF are less compared to 17.8% which was in Uganda [4]. Moreover, late parasitological failures observed in this study are higher compared to what was reported in Ethiopia by Kinfu et al. and Mekonnen et al., in which LPF of 0.0% and 4.5%, respectively, were observed after 28 days follow following treatment with ALU [20, 21]. In addition, the observed LPF is much lower compared 32.9% that was reported in Uganda [4]. The observed differences in these studies could be due to endemicity of malaria in the respective study areas and nature of the study. Most of the studies were efficacy studies in which community health workers were deployed work with parents and guardians in making sure that all children take their medicines as prescribed.

The WHO recommends the use of microscopy as a golden standard for diagnosis of malaria in middle and low income countries. In this study, microscopy was also used to assess the performance of anti-malarial drugs. Using microscopy, the observed adequate clinical and parasitological response (ACPR) were 83.3% in children in the Artefan and 77.3% in the Coartem arms. PCR uncorrected cure rate were > 80% for Artefan and 75% for Coartem. These results indicate that both generic Artefan and innovator’s product Coartem are effective for the treatment of uncomplicated malaria in children.

High adequate clinical and parasitological responses and malaria cure rate of ALU have been reported in different therapeutic efficacy studies. For instance, 100% adequate clinical and parasitological response were reported in South West Ethiopia in children under 5 years of age (23), and very low (45.4%) in Uganda [4]. PCR uncorrected cure rate were also reported to be very high (98.8%) in northwest Ethiopia [22] and south west Ethiopia (96.3%) [23]. The ability of artemisinin to clear the biomass of Plasmodium within short hours of treatment and prevention of maturation of the gametocytes by the partner drug (lumefantrine) offer the maximum performance of ALU.

Based on mid upper arm circumference (MUAC), most of the participants in this study had good nutrition status. The difference in clearing malaria parasites between those with poor nutrition status and good nutrition status could not be established. Studies have shown conflicting conclusions on the role of nutrition in relation to malaria parasite clearance with some linking poor parasite clearance and malaria morbidity and mortality with poor nutrition status [24, 25], while other studies reporting negative association between parasite clearance with malaria-induced morbidity and mortality with poor nutrition status [26, 27]. Nevertheless, emphasis must be put on taking food stuffs which are rich in iron in order to minimize incidences of anaemia in children suffering from malaria [28].

The limitation of this study is that it was designed to mimic the routine standard of care for management of uncomplicated malaria in Tanzania. In this approach, patients are given anti-malarial drugs with instruction to take the prescribed doses at home. Therefore, information on the parasite density during day 1 and day 2 after drug administration were not collected. In addition, adherence to the prescribed drugs was based on self-reports from patients, parents or guardians. Also, microscopy instead of molecular techniques, such PCR, was used to identify *Plasmodium* species. Therefore, the reported cure rate in both arms of the study are PCR uncorrected.

## Conclusion

This study revealed that Artefan as generic anti-malarial drug containing artemether and lumefantrine is equivalent to the innovator’s product Coartem in the management of uncomplicated malaria in Coast region of Tanzania. Due to low cost and availability, the use of Artefan as the generic drug is advantageous in comparison to the relatively expensive innovator`s drug such as Coartem. Therefore, the findings of this study support continuing use of generic anti-malarial drugs in the management of uncomplicated malaria in children.
